# Endocrine Therapy for the Functional Recovery of Spinal Cord Injury

**DOI:** 10.3389/fnins.2020.590570

**Published:** 2020-12-17

**Authors:** Hui Wang, Wen-xian Zhou, Jin-feng Huang, Xuan-qi Zheng, Hai-jun Tian, Bin Wang, Wei-li Fu, Ai-min Wu

**Affiliations:** ^1^Zhejiang Provincial Key Laboratory of Orthopaedics, Department of Orthopaedics, The Second Affiliated Hospital and Yuying Children’s Hospital of Wenzhou Medical University, Wenzhou, China; ^2^The Second School of Medicine, Wenzhou Medical University, Wenzhou, China; ^3^Shanghai Key Laboratory of Orthopaedic Implants, Department of Orthopaedic Surgery, Shanghai Ninth People’s Hospital, Shanghai Jiao Tong University School of Medicine, Shanghai, China; ^4^Department of Sports Medicine and Adult Reconstruction Surgery, Nanjing Drum Tower Hospital, The Affiliated Hospital of Nanjing University Medical School, Nanjing, China; ^5^Department of Orthopaedics, West China Hospital, Sichuan University, Chengdu, China

**Keywords:** spinal cord injury, endocrine hormone, thyroid hormones, basic fibroblast growth factor, erythropoietin, testosterone, estrogen

## Abstract

Spinal cord injury (SCI) is a major cause of physical disability and leads to patient dissatisfaction with their quality of life. Patients with SCI usually exhibit severe clinical symptoms, including sensory and motor dysfunction below the injured levels, paraplegia, quadriplegia and urinary retention, which can exacerbate the substantial medical and social burdens. The major pathological change observed in SCI is inflammatory reaction, which induces demyelination, axonal degeneration, and the apoptosis and necrosis of neurons. Traditional medical treatments are mainly focused on the recovery of motor function and prevention of complications. To date, numerous studies have been conducted to explore the cellular and molecular mechanism of SCI and have proposed lots of effective treatments, but the clinical applications are still limited due to the complex pathogenesis and poor prognosis after SCI. Endocrine hormones are kinds of molecules that are synthesized by specialized endocrine organs and can participate in the regulation of multiple physiological activities, and their protective effects on several disorders have been widely discussed. In addition, many studies have identified that endocrine hormones can promote nerve regeneration and functional recovery in individuals with central nervous system diseases. Therefore, studies investigating the clinical applications of endocrine hormones as treatments for SCI are necessary. In this review, we described the neuroprotective roles of several endocrine hormones in SCI; endocrine hormone administration reduces cell death and promotes functional repair after SCI. We also proposed novel therapies for SCI.

## Introduction

Spinal cord injury (SCI) is a devastating nerve injury caused by severe trauma to the spinal cord, leading to temporary or permanent somatosensory, and motor dysfunction (Tohda and Kuboyama, 2011). The majority of SCI cases are caused by a sudden traumatic lesion of neural elements in spine (Alizadeh et al., 2019), and it is common for SCI patients to eventually develop severe disability, which affects both the quality of life of the patients and their family members and imposes a substantial burden on society.

**FIGURE 1:**
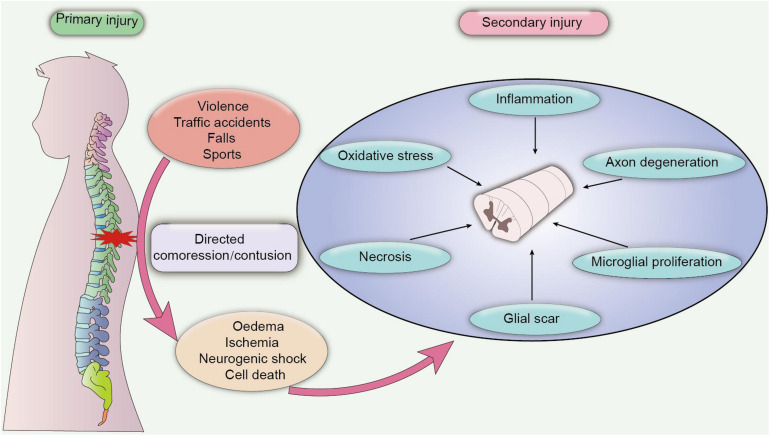
Pathological process of SCI. Primary SCI is caused by a directed compression or contusion of the spinal cord, leading to acute ischaemia and oedema of the damaged tissue. Primary SCI is difficult to heal, and the pathological damage after the injury may persist for a long time, which eventually progresses into secondary SCI. Secondary SCI is caused by chronic inflammation and cytotoxic factors. In the chronic phase, inflammatory damage and cytotoxic effects induce extensive pathological damage in contused tissues, including axon degeneration, necrosis, microglial proliferation and the formation of a glial scar, which further exacerbates neuronal dysfunction and leads to adverse clinical outcomes.

According to the severity of clinical symptoms, SCI can be divided into complete injury and incomplete injury. Complete SCI is characterized by the loss of sensory and motor function below the injured section, while incomplete SCI refers to the incomplete loss of motion and sensory function of the lowest sacral segment below the injury plane. In addition, contusion of spinal segments elicits distinct disabilities. For instance, quadriplegia occurs when the cervical vertebra segment is injured, and paraplegia occurs when the lower thoracic segment is injured (Wilson et al., 2012). The clinical outcome of SCI is strongly associated with the degree and position of spinal injury, as patients with complete SCI have a poor prognosis with severe sensory and motor dysfunction, also, these patients are refractory to electrical nerve stimulation therapies or traditional pharmacological therapies (Komatsu et al., 2019). On the contrary, patients with incomplete SCI exhibit better recovery of locomotor function after neurotrophic treatment, with highest recovery rate observed during the first 3 months after injury (Waters et al., 1994). Additionally, SCI also can be classified into primary injury and secondary injury, based on the pathological nature of the damage. Primary injury to the spinal cord is caused by the initial direct compression or contusion of the spinal cord, which results in ischaemia and oedema of the compressed cord. Secondary injury is characterized as chronic damage caused by several pathological changes after injury, leading to the activation of the inflammatory response, oxidative stress, apoptosis, and necrosis of neurons (Zhou et al., 2019; Figure 1). Mechanistically, secondary injury induces the release of a cascade of neurotoxic factors in the lesion site, which not only aggravate the inflammatory response and cell death of damaged neurons but also elicit the formation of a glial scar, demyelination and axon degeneration, contributing to the deteriorating clinical outcome (Bighinati et al., 2020).

During the early stage of injury, primary mechanical compression of the spinal cord causes oedema, ischaemia and hypoxia of adjacent tissues and stimulates immune activity that can aggravate tissue damage (Rolls et al., 2009). The persistent stress on the spinal cord further instigates excessive bleeding and neurogenic shock, causing the loss of vascular tone and hemangiectasis, which results in decreased resistance of surrounding blood vessels, vascular compromise, inflammation and immune responses. Moreover, vasospasm caused by tissue ischaemia can disrupt blood flow to the spinal cord and induce various lesions in both white matter and gray matter, including damaged myelin, axonal swelling and decreased Hematoxylin-Eosin staining (Tator and Koyanagi, 1997). Tissue ischaemia then triggers the death of a large number of cells and a cascade of cytotoxic reactions, such as iron imbalance, the production of inflammatory chemokines and free radicals (Agrawal and Fehlings, 1996), and stimulates the activation of microglial cells and caspase proteins, which can also promote the apoptosis and necrosis of nerve cells (Beattie et al., 2000). Inflammation plays an important role in secondary SCI. Numerous inflammatory cells, such as microglia, macrophages, T-lymphocytes and astrocytes, are activated in the injured areas after SCI and produce large amounts of proinflammatory cytokines and chemokines (Popovich et al., 1997), resulting in severe cell damage, axon degeneration, and demyelination (Samantaray et al., 2016b). Fortunately, the spontaneous recovery of neural function is elicited to impede neurotoxic effects after injury (Cassidy and Cramer, 2017), and macrophages and astrocytes are activated to produce neurotrophic factors that promote nerve regeneration. In addition, a mass of glial scar tissue is formed to block the spread of inflammation and cytotoxic factors, which also has been identified as a protective response to reduce secondary injury (Rolls et al., 2009). However, endogenous neurogenesis is incomplete, and the proliferation of oligodendrocytes and remyelination is inhibited due to inflammation and ischaemia (Shultz et al., 2017; Zhu et al., 2020). Additionally, excess formation of glial scar tissue is considered the main factor inhibiting axon extension and myelination, which is also the main factor hindering the progression of current research.

Spinal cord injury is a long-standing pathological state and primary SCI tends to progress into chronic SCI. Both primary and secondary injuries stimulate a series of cellular and molecular processes that subsequently induce the accumulation of proinflammatory chemokines and cytotoxic factors at injured site and cause significant sensorimotor dysfunction, neuromuscular injury, and muscle loss at the injured site (Otzel et al., 2018). For instance, patients with chronic SCI usually suffer severe complications, such as muscle atrophy (Castro et al., 1999; Gorgey and Dudley, 2007), pressure ulcers, urinary retention, erectile dysfunction (D’Andrea et al., 2020), increased fat mass and insulin resistance (Spungen et al., 2003; Gorgey et al., 2017), and they are vulnerable to develop cardiovascular disease (Gorgey et al., 2017). Among these complications, muscle atrophy and fat infiltration in the lower extremities are common secondary complications caused by the deposition of adipose tissue into skeletal muscle, and the magnitude of lower extremity muscle atrophy and fat infiltration is based on the severity of SCI. Patients with complete SCI have a lower muscle cross-sectional area and density than patients with incomplete SCI, which may be attributed to the different functional improvements during the chronic stage of injury (Moore et al., 2015). In summary, the deposition of a fat mass into skeletal muscle leads to a chronic inflammatory response and dyskinesia (Elder et al., 2004), resulting in the increased early death of and medical care required by patients with SCI (Garshick et al., 2005).

Primary SCI caused by direct mechanical damage to the spinal cord is irreversible, with little response to standard therapies; however, secondary SCI is caused by subsequent pathological changes after injury and can worsen over time. Understanding of the pathological processes and type of damage caused by SCI is urgent and may provide potential for clinical treatment. The common treatments of SCI are mainly focused on nerve regeneration and anti-inflammatory agents. According to Liu et al. (2020), an apocynin treatment facilitates the recovery of forelimb motor function by inhibiting microglial activation. Jiang et al. reported that the suppression of the NLPR3 inflammasome reduces mitochondrial dysfunction and inflammatory reactions by inhibiting neutrophil invasion to promote neurological recovery after SCI (Jiang et al., 2017). Although numerous *in vivo* and *in vitro* studies have explored the cellular and molecular mechanisms of SCI, and several therapies have been identified as achieving good therapeutic effects on SCI animal models, the number of efficient treatments applied in the clinic is limited (Dietz and Curt, 2006). To date, a consistent consensus on the therapy for neurological impairments in patients with SCI has not been achieved. Methylprednisone sodium succinate (MPSS) has been widely used as a standard drug to treat SCI, but the utility of MPSS in terms of a clinical therapeutic effect remains controversial due to the severe adverse events. Additionally, several pharmacological approaches, such as anti-inflammatory agents and nerve growth factors, have been developed to inhibit the inflammatory response and cell death in injured nervous tissues *in vivo* and *in vitro* studies (Wada et al., 2018; Jiao et al., 2020; Zhang et al., 2020), but beneficial neuroprotection in clinical trials has not yet been achieved because of the discrepancy with the basic principle of drug administration.

In recent years, several studies have reported that in addition to the impaired nerve and locomotor functions, patients with SCI usually exhibit significantly lower levels of endocrine hormones (Durga et al., 2011; Gorgey et al., 2017; Otzel et al., 2018; Dirlikov et al., 2019), both males and females with SCI showed a prevalence of low level of serum total testosterone (Dirlikov et al., 2019). Besides, impaired endocrine function further leads to metabolic anomalies and the development of depressive symptoms, accompanied with significant increase of body weight and life dissatisfaction. While the administration of exogenous hormones improves both reduced hormone levels and impaired neurological function in SCI patients (Fernandez et al., 2004; Gazula et al., 2004; Byers et al., 2012; Dell’Acqua et al., 2012; Yarrow et al., 2014; Bielecki et al., 2016), and the activity-based physical rehabilitation accompanied with testosterone administration can significantly promote muscular recovery and limited functional improvement after SCI. Endogenous hormones refer to proteins, peptides and lipids secreted by various endocrine cells. In the physical process, endogenous hormones secreted into the circulation target various organs to regulate their physiological functions. Adjuvant hormone therapies have been commonly applied to patients with various diseases, including women with breast cancer (Ramchand et al., 2019), postmenopausal women with reduced bone mass (Khosla and Monroe, 2018), and individuals with hypothyroidism (Biondi and Wartofsky, 2014; de Carvalho et al., 2018), hypocalcaemia (Maxwell et al., 2017; Streeten et al., 2017), and growth retardation (Wakeling et al., 2017). Moreover, brain tissues can spontaneously produce various hormones under physiological and pathological conditions to promote axon extension and myelination and to ameliorate inflammatory and immunological responses (Sribnick et al., 2006; Arevalo et al., 2015), and the magnitude of nerve injury after acute neurotic insult can be influenced by altered levels of endocrine hormone. For instance, the brain-derived oestradiol is induced in the injured tissue to regulate the behavior of synaptogenesis and synaptic plasticity (Arevalo et al., 2015), which is favorable for the rehabilitation of impaired neurological function. Considering the neuroprotection of endocrine hormones in several neurotic insults and potential for the treatment of SCI, profound discussions of the role of endocrine hormones in neurodegeneration and SCI are urgent. Therefore, this review aims to analyze the neuroprotective functions of several endocrine hormones in SCI and discuss their feasibility for application in future studies. In this review, we discuss the neuroprotective effects of several common endocrine hormones, including estrogen, testosterone, erythropoietin (EPO), basic fibroblast growth factor (bFGF), and thyroid hormones (THs), on SCI and some degenerative disease of the central nervous system (CNS). In addition to the traditional roles of these hormones in regulating homeostasis, each hormone can promote neuronal regrowth and the recovery of locomotor function. In addition, we also analyze the purported mechanisms of each endocrine hormone involved in the improvement of SCI, including promoting the differentiation and maturation of oligodendrocytes, increasing axon regeneration and action potential firing, and inhibiting the inflammatory response. We propose that the administration of endogenous hormones may represent a promising therapy for devastating SCI in the future.

## Estrogen

### The Neuroprotective Role of Estrogen in Neurodegeneration

Estrogen is one steroid hormone that is mainly synthesized by the ovaries of female mammals, and it is also be produced in small amounts by the testicular tissue. Estrogen is a versatile hormone that can regulate physiological processes by binding to Estrogen receptors (ERs) on the surface of the cell membrane.

Estrogen has been traditionally identified as a reproductive hormone that is involved in the development of the female reproductive system and maintaining secondary sexual characteristics in humans. However, in recent years, the neuroprotective effects of estrogen have been widely discussed, and similar to brain-derived neuroprotective factor (BDNF) which is synthesized by the brain tissue, estrogen ameliorates several neurodegenerative diseases to maintain cerebral homeostasis (Arevalo et al., 2015). Estrogen has been reported to reduce oxidative stress and cell death in individuals with different neuropathic conditions, and can ameliorate the symptoms of Parkinson’s disease (PD), Alzheimer’s disease and cerebrovascular stroke (Corvino et al., 2015). Moreover, estrogen administration increases the expression of the anti-apoptotic factor Bcl-2 and the neuroprotective gene BDNF in trimethyltin-treated rats, in which hippocampal neurodegeneration is widely localized in the pyramidal layer of the CA1 and CA3 hippocampal subfields (Wang et al., 2003). Estrogen also activates multiple neuroprotective signaling cascades, such as the extracellular signal-regulated kinase (ERK) and phosphoinositide 3-kinase (PI3K) signaling pathways, to interact with other neuroprotective molecules, including the activation of anti-apoptotic gene BCL-2, and restore the impaired nerve function (Arevalo et al., 2015).

### Estrogen and Estrogen Receptors-Mediated Neuroprotective Signaling

**FIGURE 2:**
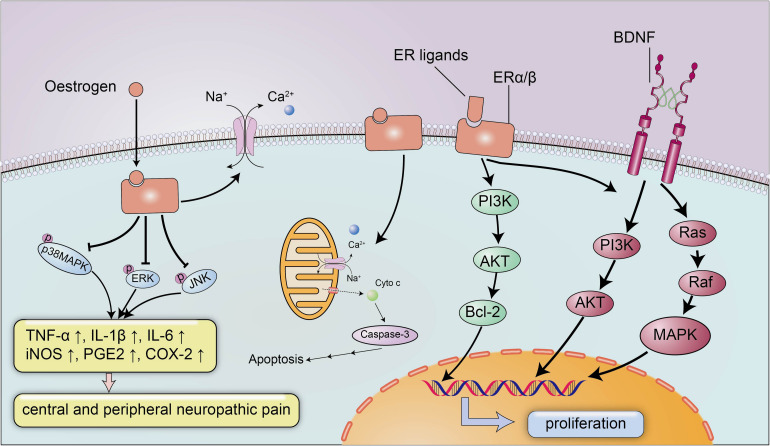
The neuroprotective signaling pathways of estrogen and estrogen receptors. Estrogen exerts its neuroprotective effects mainly by binding to estrogen receptors ERα/β. Estrogen and ER ligands can enhance the expression of anti-apoptotic genes and brain-derived neurotrophic factors and suppressed the expression of pro-apoptotic genes and pro-inflammatory molecules. Estrogen can inhibit the expression of inflammatory factors TNF-α, IL-1β, IL-6, iNOS, PGE2, and COX-2 by reducing the levels of p-p38 MAPK, p-ERK and p-JNK, to ameliorate central and peripheral neuropathic pain. In addition, estrogen can also maintain membrane integrity and inhibit apoptosis by preventing cytochrome c and calpain influx into the injured area.

The neuroprotective effect of estrogen has been widely recognized, and estrogen plays an important role in brain development and CNS inflammation mainly by binding to ERs ERα and Erβ (Figure 2). ERs can promote the development of the brain tissue and are indispensable for the migration and survival of neurons. Mice lacking ERβ display a block of the maturation of glial cells and deteriorative necrosis and apoptosis in neurons, with smaller brain sizes and fewer neurons detected in the cortex during the embryonic stage (Lee et al., 2012). Moreover, the expression of ERα and ERβ decreases after SCI in conjunction with severe neural defects and cell death (Tiwari-Woodruff et al., 2007), and selected doses of ERα and ERβ ligands appear to reverse neurodegeneration and exert a neuroprotective effect on animals with experimental autoimmune encephalomyelitis (EAE), a multiple sclerosis model characterized by CNS inflammation and neurodegeneration, by reducing demyelination and axonal loss in the white matter and in cortical neurons. Nevertheless, the neuroprotective effects of ERα/ERβ ligands may not depend on the anti-inflammatory properties of estrogen, and a treatment with ERα ligands completely abrogates EAE at the onset and throughout the disease course, while a treatment with ERβ ligands only induces clinical recovery during the chronic stage of the disease (Scharfman and MacLusky, 2006). The weak association between the anti-inflammatory effects and ERα/ERβ ligands suggested the potential use of ERα and ERβ ligands to treat several neurodegenerative diseases with a small inflammatory component, and the administration of anti-inflammatory agents in combination with ERα and ERβ ligands was proposed as a treatment for significant inflammatory diseases.

Additionally, estrogen activates BDNF signaling in the brain to exert neurotrophic effects (Figure 2; Almeida et al., 2005). BDNF functions as a neuroprotective factor in the CNS under both physiological and pathological conditions. BDNF reduces neuron apoptosis and necrosis induced by glutamate excitotoxicity and improves the morphology of hippocampal neurons by activating the PI3K and Ras/MAPK pathways (Ray and Banik, 2003). Moreover, estrogen induces the expression of BDNF, which in turn increases synaptogenesis and neuropeptide Y (NPY) synthesis in hippocampal neurons; NPY is a neuromodulator that directly exerts a neuroprotective effect to inhibit apoptosis (Almeida et al., 2005). In addition to the estrogen-BDNF-NPY molecular cascade regulating estrogen-related neuroprotection in several neurodegenerative diseases, estrogen can also attenuate neuronal apoptosis by suppressing excessive calpain activation after SCI. A supplementary study identified the possible mechanism by which estrogen inhibits cell death (Sribnick et al., 2006): the calpain content in the lesion was increased approximately three-fold after SCI, followed by significant increases in caspase-3 activity, cytochrome c levels and apoptosis compared with the sham-operated group, and the increased calpain activity in the lesion is a pathological reaction commonly detected after SCI that can induce membrane instability and cell death (Yue et al., 2005). However, estrogen inhibits the activation of calpain by preventing calpain influx into the injured area to maintain membrane integrity and inhibit apoptosis (Sribnick et al., 2006).

Estrogen has versatile neurological functions. The spontaneous synthesis of oestradiol and several neuroprotective factors is induced in damaged sites of the CNS to alleviate inflammation and cell destruction (Arevalo et al., 2015), while various nerve defects, such as neuronal loss, hippocampal neurodegeneration, cognitive dysfunction and Abeta plaque formation, are elicited in individuals with a decreased estrogen level (Azcoitia et al., 1999; Wang et al., 2003; Lee et al., 2018; Cui et al., 2019). Female rodents with ovariectomy display increased β amyloid peptide (Aβ) accumulation and lower oestradiol level in the CNS, and tend to develop severe disorders associated with neurodegeneration (Azcoitia et al., 1999). Meanwhile, the expression of the neuroprotective factor aromatase, which is presumed to increase the estrogen levels in damaged neurons, is decreased in animal models with nerve injury (Cui et al., 2019). Nevertheless, the addition of exogenous estrogen to ovariectomized rats reduces toxicity-induced cell death and the loss of hippocampal neurons in the damaged brain, and promotes axon extension and synaptogenesis, which ultimately alters the neuronal vulnerability to excitotoxicity (Wang et al., 2003; Lee et al., 2018).

### The Neuroprotective Role of Estrogen in SCI

Based on the neuroprotective effects of estrogen, many studies have explored the therapeutic effects of estrogen on SCI. As shown in the study by Lee et al., the administration of a single dose of 17β-oestradiol (100 or 300 μg/kg) to rats ameliorated neuropathic pain, including mechanical allodynia and thermal hyperalgesia, in the late phase after SCI compared to the littermates (Hains and Waxman, 2006). The phosphorylation of p38 mitogen-activated protein (MAP) kinase (p38 MAPK), ERK, and JNK play pivotal roles in the induction and persistence of central and peripheral neuralgia (Samantaray et al., 2016a), and 17β-oestradiol reduces the levels of p-p38 MAPK, p-ERK, and p-JNK by inhibiting the activation of microglia and astrocytes to relieve neuralgia (Figure 2; Hains and Waxman, 2006). According to Samantaray et al., the administration of a low dose of estrogen improves disruptions in locomotor function and axonal and myelin loss in rodent models of acute SCI, with synergistic interactions to decrease cell death by stimulating angiogenesis and reducing the inflammatory response and glial reactivity (Samantaray et al., 2016b; Farag et al., 2019). In a study examining kainic acid (KA)-induced SCI, the oestradiol treatment did not improve the damaged cerebellar histoarchitecture after injury, however, the conversion from an anomalous cell morphology to a normal architecture was observed by using conventional or immunofluorescence staining, which appears to illustrate the anti-inflammatory effect of oestradiol on ameliorating neurotoxicity in cerebellar layers (Amantea et al., 2005).

### Limitations and Challenges

Generally, the protective effects of estrogen in neurodegeneration and SCI animal models are remarkable, while controversies still exist in individuals with SCI. Greenwald et al. reported that no gender-associated differences was found in the Functional Independence Measure (FIM) motor scores and American Spinal Injury Association (ASIA) scores on acute-care admission and rehabilitation discharge in SCI patients (Greenwald et al., 2001). While a retrospective study disclosed that men obtained a larger improvement of 5.3 in Spinal Cord Ability Ruler (SCAR) score as compared to women (Bach Jonsson et al., 2019). Although co-morbidities, therapeutic approaches, length-of-stay in the acute care unit, mortality, and discharge disposition were reported to be similar in men and women with SCI, and both men (44.7%) and women (52.9%) were similar in the development of post-SCI secondary complications, research had found that female patients were sensitivity to suffer from psychiatric complications and deep venous thrombosis (Furlan et al., 2005). In contrast, a study involving 14433 people reported that women had more significantly natural neurologic recovery than men, indicating that gender differences in SCI were existed in several areas (Sipski et al., 2004). Actually, whether or not SCI patients have gender-related differences in the neurologic rehabilitation is still unclear, and the administration of high doses of estrogen have severe safety concerns, such as the risks of deep venous thrombosis and coronary heart disease, which hamper the utility of clinical application.

Recently, novel drug delivery systems have exhibited more favorable treatment effects in SCI animal models. By the focal delivery of estrogen to the injured spinal cord, desired therapeutic effect may be achieved while systemic levels of estrogen can be kept in a normal physiological range. For instance, Cox et al. (2015) designed nanoparticle drug delivery systems that can locally release estrogen into damaged spinal cord tissue, and the tissue level of estrogen were double those detected in the plasma, which indicated focal release. Those new drug delivery approaches may provide potential for the safety applications of pleiotropic estrogen in SCI patients, and more persuasive clinical data are indispensable for the validation in humans.

## Testosterone

### SCI With Low Testosterone Levels

Studies have reported that approximately 50% men suffer from lower levels of testosterone after SCI, who are usually accompanied by significant impairments in muscle mass, locomotor function and hypogonadism (Otzel et al., 2018), and patients with chronic SCI are more likely to complain about erectile dysfunction, decreased sexual function and life satisfaction (D’Andrea et al., 2020). Additionally, the testosterone level is associated with the severity of SCI, as a testosterone deficiency is more prevalent in patients with a complete loss of motor function than in patients with an incomplete loss of motor function (43.3% VS 16.8%), and patients who require narcotic pain medications to relieve pain have lower serum testosterone levels (Durga et al., 2011). Furthermore, a lower testosterone level is associated with increased fat mass and body mass index (BMI; Abilmona et al., 2019), and patients with a low serum testosterone level tend to develop severe chronic metabolic disorders, such as type 2 diabetes and cardiovascular disease (Gorgey et al., 2017).

An insufficient level of testosterone has also been observed in rat models of chronic SCI, in which the serum testosterone level was 49–55% lower than in sham animals at 4 weeks after injury, and the levator ani and bulbocavernosus (LABC) muscle mass was also 13% lower in the SCI group than in controls (Yarrow et al., 2017). However, the loss of muscle mass and body composition can be repaired after androgen treatment in SCI models (Gregory et al., 2003), and a testosterone treatment protected the quadriceps muscles from a decrease in the dendritic length of motoneurons caused by SCI-induced impairments (Byers et al., 2012). The increased muscle fiber cross sectional area (CSA) was also discovered in chronic SCI patients treated with testosterone, and testosterone replacement therapy couple with evoked resistance training in SCI patients deserved a significant 27.5% increase in fiber CSA when compared with testosterone replacement therapy only, demonstrating the muscular-protective effects of testosterone in SCI (Gorgey et al., 2020b). Therefore, testosterone may function as a neuroprotective factor in several nervous system diseases.

### Androgen Is Neuroprotective

Androgen is an anabolic protein that plays a pivotal role in the development of the body composition and the quality of life. Androgen promotes the synthesis of muscle, protein and bone, and increases lean body mass in males after SCI (Nightingale et al., 2018); it can also attenuate the accumulation of ectopic fat and insulin tolerance when used to treat several metabolic diseases (Gorgey et al., 2017). A study reported the dose-dependent effect of testosterone on protecting against bone and muscle loss in rodents with chronic SCI, as low-dose testosterone-enanthate (2.0 mg/week) slightly ameliorated cancellous bone and LABC muscle loss with no effect on the hindlimb muscle mass, and high-dose testosterone-enanthate (7.0 mg/week) increased cancellous bone and LABC muscle mass (Yarrow et al., 2014). In addition, for chronic SCI patients, testosterone replacement therapy also seems to have robust effects in the recovery of muscle mass, and resistance training combined with low-dose testosterone replacement therapy resulted in significant hypertrophy on the adjacent untrained glutei muscles, as compared to patients with testosterone replacement therapy only (Gorgey et al., 2020a, b).

Since skeletal muscle function is governed by somatic motor neurons, the degeneration of the dendrites and axons of neurons in lesions after SCI represent the dominant factors contributing to an insufficient muscle mass and skeletal muscle atrophy, but androgen can preserve both muscle mass and motoneuron dendrites by suppressing the degeneration of dendrites (Byers et al., 2012). Additionally, the administration of testosterone increases axon regeneration in the lesion to mitigate dendritic atrophy (Byers et al., 2012), with a neuroprotective effect on activating motoneuron and reversing impaired locomotor function (Gazula et al., 2004). Testosterone treatments reverse dendritic atrophy after SCI, increase the number of spared or sprouting axons in damaged areas and promote axon sparing processes to prevent the degeneration of the associated muscle mass and fibers (Byers et al., 2012). Furthermore, testosterone also increases the proliferation and differentiation of oligodendrocyte precursors in the hippocampus to promote myelination (Bielecki et al., 2016). Hussain et al. (2013) reported that testosterone stimulates myelin sheath regeneration and repair by activating androgen receptor in the brain and inhibiting the proliferation of microglia and astrocytes to reduce neuroinflammatory responses.

The neuroprotective effect of testosterone on nerve diseases has mainly been confirmed by the activation of androgen receptors in neurons throughout the CNS, and the activated androgenic pathways are involved in the regulation of multiple functional proteins that can inhibit inflammatory and oxidation reactions to improve neuroprotection and attenuate nerve degeneration. Mechanistically, testosterone passes through the blood-brain barrier and binds to androgen receptors in neurons to influence neuronal differentiation and growth (Iqbal et al., 1983). Testosterone administration increases the volume of gray matter in right frontal cortex, reverses gray matter atrophy in individuals with several inflammatory and neurodegenerative diseases, such as multiple sclerosis (Kurth et al., 2014), and decreases the expression of apoptosis-related proteins, such as caspase proteins, in response to ischaemia-reperfusion injury of the spinal cord to reduce neuronal apoptosis (Gurer et al., 2015). However, the neuroprotective and neurotherapeutic effects of testosterone are significantly inhibited by androgen receptor antagonists. Therefore, the beneficial effects of a testosterone treatment on axon regeneration and motoneuron survival are potentially attributed to the receptor-mediated activation of hormone signaling pathways to exert neuroprotective and neurotherapeutic effects, and the possible mechanism underlying the neuroprotective effects of testosterone requires further study for better clinical applications in the future.

## Erythropoietin

**FIGURE 3:**
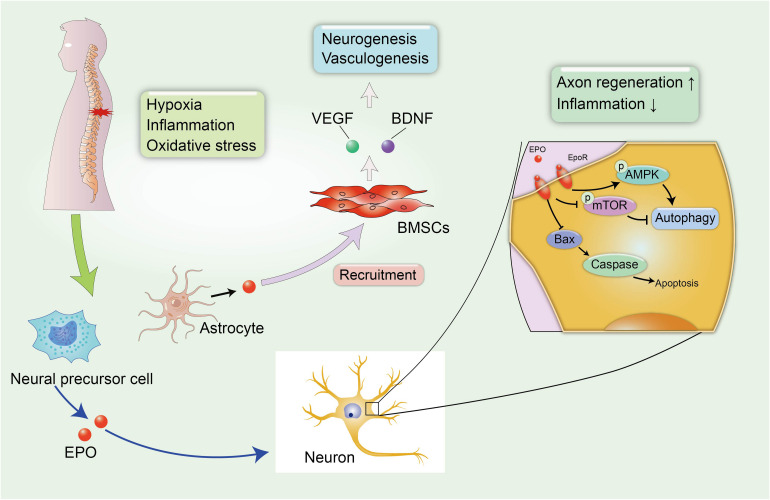
EPO-related neuroprotective effects in SCI. The secondary SCI induces abnormal microenvironment and deteriorates hypoxic and inflammatory condition in damaged tissues. Fortunately, neural precursor cells and astrocytes can spontaneously synthesize EPO under hypoxic conditions to alleviate neural insult and demyelination. In addition, EPO induces the recruitment of BMSCs to the injured spinal cord, leading to the increased expression of VEGF and BDNF, which can promote neurogenesis and vasculogenesis.

Erythropoietin is a pleiotropic hormone with remarkable neuroprotective effect. EPO also known as a hematopoietic growth factor is secreted from the kidney and is named for its ability to promote the formation of red blood cells. The traditional application of EPO is mainly focused on the treatment of anemia caused by chronic renal failure (CRF) or an advanced malignant disease (Winearls et al., 1986). Recently, several reports have shown that EPO protein and EPO receptors (EpoRs) were abundantly expressed by human glial cells and neurons in the spinal cord and brain at the stage of fetal development during pregnancy, and EPO can be activated under hypoxic conditions, with nearly a two-fold increase in the expression of the EPO mRNA in neurons to exert neuroprotective effect (Figure 3; Juul et al., 1998). However, researchers have not yet clearly determined how EPO ameliorates the pathological process in the microenvironment of the CNS, and a clear understanding of the protective effects of EPO on SCI and other neurodegenerative diseases is urgently needed.

## Epo Is Neuroprotective

A large number of studies have reported the neuroprotective effect of EPO in neurological disorders. Li et al. (2017) reported that the administration of EPO promoted the recruitment of BMSCs to the injured spinal cord, leading to the increased expression of endothelial growth factor (VEGF) and BDNF. VEGF is known for its role in angiogenesis, because it increases the microvessel density and vascular permeability in damaged areas and can improve locomotor function (Patel et al., 2009). The role of EPO in promoting the synthesis of VEGF and BDNF has been identified as promoting the recovery of neurophysiological function after SCI (Figure 3). In addition, EPO promotes neurogenesis and oligodendrocyte survival in the hippocampus by activating several regulated signaling cascades, such as neurotrophin and CREB, to improve cognitive dysfunction (Tiwari et al., 2019). Additionally, neural precursor cells release endogenous EPO, which gradually accumulates at the edge of lesion to attenuate post-traumatic neuroinflammation and myelin loss, and improves the microenvironment around the spinal cord to create a suitable condition that is favorable for axon regeneration (Carelli et al., 2017). A similar result was also confirmed in rodent models of SCI treated with recombinant human EPO (rhEPO), as the addition of 1000 and 3000 units of rhEPO significantly reduced inflammation after SCI and induced a significant recovery of motor function at 7 days after injury. Meanwhile, a daily dose of 500 units of rhEPO administered for 7 days did not produce an improvement compared with controls, and the amelioration of neurological function by rhEPO administration was postulated to explain the recovery of blood flow after injury (Gorio et al., 2002). Cerri et al. (2012) also reported that rhEPO improved the rehabilitation of motor function in the lower extremities and promoted the regeneration of the myelin sheath at the injured site over time to stimulate the transmission of action potentials. Mechanistically, EPO is involved in increasing cell survival in the presence of several pathological conditions, including apoptosis and impaired autophagy (Jang et al., 2016; Wang et al., 2018; Zhou et al., 2019). Apoptosis is a physiological cell process in which unwanted or abnormal cells are eliminated by programmed cell death, and it is one of the hazards verified in secondary SCI. Animal models with SCI display excessive activation of apoptosis-related molecular mechanisms, including reduced levels of the anti-apoptotic protein B-cell lymphoma-2 (Bcl-2) and increased levels of BCL2-associated X protein (Bax) and cleaved caspase-3 (Zhou et al., 2019), which are major contributors to neurological impairments. However, the combination therapy including EPO and hyperbaric oxygen (HBO) exerted superior effect to EPO or HBO alone on inhibiting neuronal apoptosis in rats after SCI, resulting in a significant amelioration of demyelination reactions and myelin sheath degeneration and an increase in the proliferation and differentiation of oligodendrocyte precursors (Zhou et al., 2019).

In addition to inhibiting apoptosis and necrosis in injured neuronal tissues, EPO also participates in the mechanism regulating autophagy dysfunction. Physiological autophagy plays a pivotal role in the maintenance of physiological homeostasis. However, abnormal autophagy is pathologically induced after SCI, and the disruption of autophagy is a significant factor that exacerbates secondary injury (Kanno et al., 2009). Jang et al. (2016) discovered dysfunctional autophagy regulation in animal model of rotenone-induced PD, with significant recovery after treatment with EPO. As shown in the study by Wang et al., EPO activates autophagy in rats with SCI by activating the AMP-activated protein kinase (AMPK) signaling pathway in the injured spinal cord and decreasing the levels of mammalian target of rapamycin (TOR; Wang et al., 2018). The neuroprotective effects of EPO on autophagy dysfunction may be a novel mechanism for protecting against nerve injury and suggests its potential for use as a therapy for spinal cord contusions.

## Basic Fibroblast Growth Factor

Basic fibroblast growth factor is a member of the fibroblast growth factor (FGF) family that is involved in regulating various biological functions, including nerve regeneration, angiogenesis, the suppression of cell apoptosis and glial scar formation (Beenken and Mohammadi, 2009; Xu et al., 2016; Zhu et al., 2020). bFGF is an 18-kDa polypeptide that is mainly expressed in the mammalian brain (the pituitary and hypothalamus; Walicke, 1988; Wanaka et al., 1990; Dietrich et al., 1996), and it stimulates angiogenesis and vasodilation to promote wound healing (Huang et al., 2016; Zhang et al., 2018). Numerous studies have described the neuroprotective effect of bFGF on the functional recovery of neural deficits. Based on the results of an *in vivo* experiment, bFGF administration rescues 66–74% of neurons from death and reduces the necrosis of cholinergic neurons in the medial septum and diagonal band of Broca after axonal transection (Anderson et al., 1988). In addition, Dietrich et al. discovered that an intravenous bFGF infusion significantly decreased the death of lesioned cortical neurons and the overall contusion volume at 24 h after traumatic brain injury (TBI; Dietrich et al., 1996). TBI is an acute injury of the brain tissue that may cause severe contusive neuronal profiles and necrosis of cortical neurons, but an additional treatment with bFGF reduces the contusion volume and cell death in the pathological tissues. In fact, the spontaneous synthesis of bFGF is induced in brain tissues after CNS injury, and the increased level of bFGF at the lesion site is a hallmark of recovery in rat models of SCI.

In addition to increasing neuronal survival after SCI, bFGF can also ameliorate secondary injury to restore motor function. Rabchevsky et al. (2000) reported a significant recovery of hindlimb motor function in rats with SCI caused by a severe contusion after an intrathecal infusion of bFGF, and this restoration of movement was a time-dependent behavior that was gradually manifested within 2 and 3 weeks. Zhu et al. observed hypoxia in the microenvironment after compression-induced SCI that promoted glial scar formation and autophagy, concomitant with increased apoptosis, while the administration of embryonic neural stem cells (NSCs) expressing bFGF promoted axon extension and restored locomotor function at days 14, 30, and 60, with significantly higher scores on Basso-Beattie-Bresnahan (BBB) locomotion scale and angle of incline scores than in other groups (Zhu et al., 2020).

## Novel Biological Materials With bFGF for the Treatment of Nerve Injury

As a macromolecular protein involved in physiological processes, the systemic administration of bFGF after SCI appears to be unacceptable due to the persistence of several limitations, such as the inability to cross the blood-spinal cord barrier or the short circulating half-life that is responsible for its unstable biological activity (Ikada and Tabata, 1998; Lan et al., 2017). However, the novel combination therapy comprising biological materials and bFGF provides promising therapeutic potential. Biological materials are widely used to regenerate and restore the injured nerves because of their versatile properties and excellent biocompatibility. Biological scaffolds provide paths and channels with a permissive cellular environment that can direct axon extension and cell infiltration into the injured site, leading to neuronal regrowth and functional recovery (Thomas et al., 2013). Notably, bFGF-loaded gelatine microspheres increase neural regeneration and morphological recovery compared with free bFGF after spinal injury, with slow but not obvious burst release properties of bFGF from the site of transplantation and reduced proteolysis, and only 65% of bFGF was released from the bFGF-loaded complex after 28 days (Lan et al., 2017). Luo et al. (2018), Shi et al. (2014) described the neurotrophic effects of a biocompatible collagen scaffold and heparin-poloxamer hydrogel modified with bFGF on the treatment of SCI, which induced significant cell proliferation and inhibited apoptosis via the steady release of bFGF into the injured site to protect it from enzymatic degradation. Xu et al. (2016) also reported similar results for bFGF encapsulated in a hydrogel, with significant inhibition of neuronal apoptosis and astrocyte activation in a rat model of SCI, and a 50% increase in the PC12 cell survival rate at 24 h compared with a bFGF solution. This bFGF complex achieved satisfactory outcomes of nerve regrowth and locomotor recovery in the comparison with the bFGF solution group. Zhao et al. (2016) also reported the superiority of a novel bFGF-nanoliposome (bFGF-NL) complex in neuroprotection compared with free bFGF protein therapy in a brain injury model. The bFGF-NL complex penetrated the blood-brain barrier and accumulated in the damaged brain tissue, subsequently restoring locomotor function and neuronal plasticity, changes that were blocked by PI3K/AKT inhibitors. A combination therapy with biological materials optimizes the neuroprotective effect of bFGF on SCI, and bFGF-loaded gelatine plays a role in angiogenesis and the inhibition of cell death.

## Thyroid Hormones Promote Neuronal Proliferation and Differentiation

Thyroid hormones are important for the development of the CNS in mammals, particularly during the embryonic and fetal stages of brain development (Talhada et al., 2019). THs promote neuronal proliferation and differentiation, synaptic plasticity and myelination (Gothie et al., 2017). Fernandez et al. reported that the administration of tetraiodothyronine (T4) induced remyelination in an animal model of experimental allergic encephalomyelitis and improved the morphology of axons and the thickness of the myelin sheath to protect against nerve degeneration (Fernandez et al., 2004). A similar outcome was also reported in another study, and a triiodothyronine (T3) treatment enhanced the maintenance of nerve conduction and neuroplasticity in the lumbar spinal cord of EAE animals, contributing to maintaining the integrity of axons and myelin sheaths (Dell’Acqua et al., 2012). In addition, THs play a role in neuroprotection by stimulating the repair of damaged neurons and by increasing oligodendrocyte proliferation and axon regeneration to reduce the production of proinflammatory cytokines and cytotoxic factors (Billon et al., 2002; Dell’Acqua et al., 2012).

Studies have observed lower levels of T3 and testosterone in men with SCI (Dirlikov et al., 2019). Thyroid hormone signaling is inhibited because of the increased expression of the thyroid hormone-inactivating enzyme deiodinase 3 and decreased expression of thyroid hormone receptors (THRs) in an animal model of neuroinflammation (Fernandez et al., 2016). Nevertheless, several nerve defects, such as demyelination, re partially repaired after the administration of THs (Calza et al., 2002; Fernandez et al., 2004; Dell’Acqua et al., 2012), and 3-iodothyronamine (T1AM) also attenuate neural degeneration and impaired hind limb motor function by stimulating TAAR1 in an SCI model, which subsequently activates the apoptosis pathway (Lv et al., 2018). Therefore, the neuroprotective effect of THs should be considered because of their potential for treating serious neurodegenerative diseases.

Under physiological conditions, THs bind to nuclear receptors in target cells to regulate biological functions, and nuclear thyroid hormone receptors are widely expressed on oligodendrocytes. According to Dugas et al. (2012), T3 induces the expression of the transcription factor KLF9 by binding to nuclear THRs in oligodendrocytes, KLF9 is necessary for the differentiation of oligodendrocytes *in vitro*, and the T3-induced activation of KLF9 promotes oligodendrocyte differentiation and myelination.

Oligodendrocytes participate in cellular metabolism in the CNS and ensure the generation of myelin membrane and the integrity of axons. Oligodendrocytes provide neurotrophic support for axon growth in the microenvironment and reduce axonal injury and myelin degradation, which are very important for the maintenance and conduction of action potentials in axons (McTigue and Tripathi, 2008; Kim and Petratos, 2019; Nutma et al., 2020). The spontaneous immune response of the nervous system was activated after nerve injury, and oligodendrocytes produce several chemokines and cytokines to induce a cascade of cellular and molecular reactions, which represents a hallmark of decreased inflammation in several diseases (Peferoen et al., 2014). Furthermore, inflammation is a dominant factor preventing the non-myelinating oligodendrocytes from differentiating into myelinating oligodendrocytes and remyelination in the injured CNS, and a disruption of oligodendrocyte development is a common neuropathological finding in contusive spinal cord injuries. In fact, oligodendrocyte damage is a pivotal feature of CNS injury because it blocks the differentiation of oligodendrocyte precursor cells and induces the production of abundant cytotoxic factors, resulting in axon degeneration, demyelination, cell apoptosis, and necrosis (McTigue and Tripathi, 2008). THs are involved in regulating the maturation of oligodendrocyte precursors (Billon et al., 2002; Calza et al., 2002). THs induce the differentiation of multipotential stem cells into myelinating oligodendrocytes and promote the production of oligodendrocyte precursors, which modulate different stages of oligodendrocyte development.

## Biological Materials Implanted With Ths for Appropriate Drug Utilization

The systemic administration of T3 poses a risk of hyperthyroidism, with little efficiency in the treatment of the injured spinal cord tissue. Shultz et al. (2017) reported that the valid therapeutic range of T3 for SCI was 10–2,000 ng ml^–1^, which are much higher levels than the circulating level of T3 in patients with hyperthyroidism. A hydrogel containing T3 had the ability to locally deliver T3 to the lesion, with a completely effective dose for oligodendrocyte differentiation and maturation, as well as myelination (Shultz et al., 2017). Bighinati et al. designed an implantable biomaterial containing ibuprofen and T3 that provided long-term release of T3 at the lesion site. The local delivery of T3 by the implantable biomaterial facilitated the maturation of myelinating oligodendrocytes and remyelination in the injured spinal cord, and reduced the spontaneous release of glutamate, which was excitotoxic to neurons (Bighinati et al., 2020). Therefore, a treatment combining T3 with biological materials potentially represents a promising therapy in future studies.

## Conclusion

Spinal cord injury is the main cause of physical disability and is responsible for the overuse of medical care and increased socioeconomic burdens. The chronic, progressive loss of the myelin sheath and neuroinflammation increase the apoptosis and necrosis of neurons, which inhibit nerve regeneration and motor function recovery after SCI, and are also verified as risk factors for many refractory complications. Clinically, no effective therapy exists for the treatment of SCI. Although numerous studies have reported significant functional recovery in SCI after pharmacotherapy, the treatments for clinical application in patients with SCI are still limited. Therefore, a difficult but meaningful task is to exploit or develop a reliable and effective treatment. Endocrine hormones are regulatory molecules with the potential to treat SCI because of their non-toxic properties and ability to promote nerve regeneration. Several different hormones, including estrogen, testosterone, EPO, bFGF and THs, have been reported to play important roles in nerve regrowth and neuroprotection. With an understanding of the physical and chemical properties of those hormones and advances in endocrine therapy theory and clinical practice, the application of endocrine therapy to the treatment of SCI shows promise. At the same time, the methods for choosing the appropriate hormone or multiple hormones and for controlling the amount and balance of hormones undoubtedly require further study. In conclusion, endocrine therapy for SCI is a therapeutic approach with broad prospects that deserved further attention from researchers.

## Author Contributions

HW and A-mW designed the study. HW, X-qZ, J-fH, W-xZ, H-jT, BW, W-lF, and A-mW conducted literature collection and summary. HW and A-mW drafted the manuscript. All authors critically revised the manuscript.

## Conflict of Interest

The authors declare that the research was conducted in the absence of any commercial or financial relationships that could be construed as a potential conflict of interest.

## Abbreviations

SCI: spinal cord injury
MPSS: methylprednisolone sodium succinate
EPO: erythropoietin
bFGF: basic fibroblast growth factor
THs: thyroid hormones
CNS: central nervous system
ERs: estrogen receptors
BDNF: brain-derived neuroprotective factor
ER: estrogen receptor
NPY: neuropeptide Y
A β: β amyloid peptide
p38 MAPK: p38 mitogen-activated protein kinase
KA: kainic acid
BMI: body mass index
LABC: levator ani and bulbocavernosus
CRF: chronic renal failure
EpoR: EPO receptors
VEGF: endothelial growth factor
BDNF: brain-derived neurotrophic factor
rhEPO: recombinant human EPO
Bcl-2: B-cell lymphoma-2
Bax: BCL2-associated X protein
HBO: hyperbaric oxygen
PD: Parkinson’s disease
AMPK: AMP-activated protein kinase
TOR: target of rapamycin
FGFs: fibroblast growth factors
TBI: traumatic brain injury
NSCs: neural stem cells
bFGF-NL: bFGF-nanoliposome
THs: thyroid hormones
T3: triiodothyronine
T1AM: 3-iodothyronamine
THRs: thyroid hormone receptors
ERKs: extracellular signal-regulated kinases
PI3K: phosphoinositide 3-kinase
EAE: experimental autoimmune encephalomyelitis
LABC: levator ani and bulbocavernosus
T4: tetraiodothyronine
FIM: functional Independence Measure
ASIA: American Spinal Injury Association
SCAR: Spinal Cord Ability Ruler
CSA: cross sectional area.
